# Whole exome sequencing in relapsed or refractory childhood cancer: case series

**DOI:** 10.2478/abm-2024-0025

**Published:** 2024-09-20

**Authors:** Rungroj Thangpong, Pattarin Nuwongsri, Chupong Ittiwut, Rungnapa Ittiwut, Chureerat Phokaew, Piti Techavichit, Kanya Suphapeetiporn

**Affiliations:** Department of Pediatrics, Faculty of Medicine, Chulalongkorn University, Bangkok 10330, Thailand; Excellence Center for Genomics and Precision Medicine, King Chulalongkorn Memorial Hospital, the Thai Red Cross Society, Bangkok 10330, Thailand; Center of Excellence for Medical Genomics, Medical Genomics Cluster, Department of Pediatrics, Faculty of Medicine, Chulalongkorn University, Bangkok 10330, Thailand; Division of Hematology and Oncology, Department of Pediatrics, Faculty of Medicine, Chulalongkorn University, Bangkok 10330, Thailand

**Keywords:** exome sequencing, germline mutation, pediatric cancer, sequencing analysis, somatic mutation

## Abstract

**Background:**

The prognosis for relapsed or refractory childhood cancer is approximately 20%. Genetic alterations are one of the significant contributing factors to the prognosis of patients.

**Objective:**

To investigate the molecular profile of relapsed or refractory childhood cancers in Thai cases.

**Methods:**

The study design is a descriptive study of patients <18 years old, suspected or diagnosed of relapsed or refractory childhood cancer who underwent whole exome sequencing (WES).

**Results:**

WES was successfully performed in both the tumor and the blood or saliva samples obtained from 4 unrelated patients. Six different variants were identified in the *NCOR2*, *COL6A3*, *TP53*, and *SMAD4* genes. These alterations were found to be associated with tumor aggressiveness.

**Conclusion:**

This study is the first one to demonstrate genetic alterations by using WES in relapsed or refractory childhood cancer in Thai cases.

Childhood cancers are a heterogeneous group of diseases that lead to uncontrollable cell division caused by mutations in the proto-oncogene and the tumor suppressor gene. The prognosis is better over time with comprehensive treatment care including chemotherapy, radiation, and surgery, reaching an overall survival of approximately 80% [1]. However, about 20% of the patients develop relapsed or refractory disease with a poor prognosis despite receiving the full treatment protocol for the disease [2, 3]. Thus, complex genetic alterations may play an important role in disease progression and prognosis.

The feasibility of next–generation sequencing (NGS), including whole exome sequencing (WES), has increasingly been evaluated in cancers. The use of WES to unravel molecular profiles in patients with relapsed or refractory childhood cancers has been demonstrated. The study of molecular profiles can lead to the discovery of potentially actionable genetic alterations that provide further information about the tumor prognosis and drug targets [4].

This study reported the use of WES in the identification of genetic alterations in relapsed or refractory childhood cancers.

## Methods

### Patients

A total of 4 unrelated patients with relapsed or refractory childhood cancer with a tissue sample available at King Chulalongkorn Memorial Hospital (KCMH) were recruited in the study between January 2020 and December 2022. The patients enrolled in the study must possess accessible fresh tumor tissue at the time of diagnosis.

This study was approved by the Institutional Review Board of the Faculty of Medicine, Chulalongkorn University (IRB No. 692/65) and conducted in accordance with the Declaration of Helsinki. Written informed consent was obtained from the patients and/or their parents.

### Exome sequencing and data analysis

After obtaining informed consent, genomic DNA was isolated from the tumor samples (bone marrow in hematologic malignancy and tumor tissues in solid tumors) and from the normal samples (saliva in hematologic malignancy and peripheral blood leukocytes in solid tumors). The DNA sample was prepared as an Illumina sequencing library and in the exome capture step. The sequencing libraries were enriched with the SureSelect Human All Exon V5 Kit, Agilent. The captured libraries were sequenced using Illumina HiSeq 4000. Reads from the sequence output were aligned to the human reference genome (GRCh37) using the Burrows–Wheeler Aligner (BWA). Variants to the reference were called using the Genomic Analysis Tool Kit (GATK). The variants were annotated and filtered using the Golden Helix VarSeq analysis workflow implementing the ACMG guidelines for the interpretation of sequence variants. This includes a comparison against the gnomAD population catalog of variants in 123,136 exomes, the 1000 Genomes Project Consortium’s publication of 2,500 genomes, the NCBI ClinVar database of clinical assertions on variant’s pathogenicity and multiple lines of computational evidence on conservation and functional impact.

## Results

Four patients diagnosed with relapsed or refractory childhood cancer and with available tissue samples for DNA extraction were included and underwent WES. One was affected with hematologic malignancy (relapsed acute lymphoblastic leukemia [ALL]), and 3 had solid tumors (relapsed osteosarcoma, recurrent Wilms’ tumor, recurrent colorectal cancer [CRC]). The clinical manifestations and tumor pathology are summarized in **Table 1**. WES was performed in both tumor and normal samples from all 4 patients. Six variants were identified from the tumor samples obtained from all patients (**Table 2**). WES of the normal samples revealed no significant cancer-related variants in all cases according to the American College of Medical Genetics and Genomics/Association for Molecular Pathology (ACMG/AMP) guidelines for variant classification and the Association for Molecular Pathology, American Society of Clinical Oncology, and College of American Pathologists Recommendations [5, 6].

**Table 1.** Clinical manifestation and tissue pathology of 4 pediatric patients with relapsed or refractory cancers

**Case**	**Sex**	**Current age (year)**	**Presenting symptom**	**Histological features of tissue**	**Relapse time (month)**	**Histological feature of relapse**	**Treatment**
1	Female	14	Fever with anemia at the age of 3 years for 2 weeks	Pre-B cell ALL	- 1st bone marrow relapse: 56 - multiple CNS relapse (4 episodes) - 6^th^ bone marrow relapse: 16	Lymphoblastic leukemia	Chemotherapy Radiation
2	Female	15	A palpable mass on the left rib (no.9) at the age of 10 years for 1 month	Osteogenic osteoma	45	Osteosarcoma	Surgery
3	Male	14	Abdominal pain at the age of 12 years for 3 days	Poorly differentiated adenocarcinoma, signet ring cells	19	Poorly differentiated adenocarcinoma, signet ring cells	Chemotherapy Surgery
4	Male	6	An abdominal mass at the age of 5 years	Nephroblastoma with favorable histology	6	Nephroblastoma	Surgery Chemotherapy

**Table 2.** Summary of the WES result

**Case**	**Diagnosis**	**WES result (Classification)**
1	Relapsed acute lymphoblastic leukemia	c.5890_5906del (p.Ser1964Leufs*57) in *NCOR2*
2	Recurrent osteosarcoma	c.7475C>T (p.Ser2492Leu) in *COL6A3*
3	Recurrent colon cancer	c.757dupA (p.T253Nfs*11) (Tier III) in *TP53* c.1082G>A (p.R361H) (Tier III) in *SMAD4* c.1082G>A (p.R361H) (Tier III) in *GNAS*
4	Recurrent Wilms’ tumor	c.96+1G>C (Tier III) in *TP53*

Patient 1 was a 12-year-old girl with relapsed ALL. She presented with fever and anemic symptoms, hepatosplenomegaly, and multiple lymphadenopathies at the age of 3 years. A complete blood count (CBC) showed anemia with thrombocytopenia and hyperleukocytosis. Bone marrow examination was performed and abnormal lymphoblasts were seen, with the immunophenotype as a Pre-B cell lineage shown by flow cytometry. A chromosome study of the bone marrow sample revealed 46, XX. The diagnosis of Pre-B cell ALL was made, and chemotherapy was started. After the complete course of chemotherapy, a bone marrow examination was performed and it revealed no abnormal cells. After 56 months, the patient developed fever, anemia, and pancytopenia. Bone marrow examination showed lymphoblasts with almost 100% of cellularity. Chemotherapy for relapse was given. The patient however developed multiple episodes of central nervous system (CNS) relapse. WES performed in the bone marrow samples identified the variants, c.5890_5906del [p.(Ser2492Leu)] in the *NCOR2* gene.

Patient 2 was a 15-year-old girl with recurrent osteosarcoma. She developed a palpable mass on the left side of the ninth rib at the age of 10 years. The pathology of the excised tissues revealed osteogenic osteoma. Imaging studies showed no evidence of metastasis. The patient received adjuvant chemotherapy. About 45 months later, the patient developed pulmonary metastases in the right upper lung (RUL) by computerized tomography (CT) scan. RUL lobectomy was performed and the tissue pathology revealed osteosarcoma. WES identified the variant, c.7475C>T [p.(Ser2492Leu)] in *COL6A3*.

Patient 3 was a 14-year-old boy with recurrent colon cancer. He presented with acute right lower abdominal pain with fever and vomiting without a history of abnormal defecation at the age of 12 years. The patient underwent an exploratory laparotomy with a right hemicolectomy. A mass in the mid-transverse colon with the size of 3 cm × 3 cm was detected. The tissue pathology revealed a poorly differentiated adenocarcinoma, a type of signet ring cells with foci of neuroendocrine differentiation. The patient subsequently received chemotherapy. During the treatment, an abdominal CT scan showed multiple peritoneal nodules. The biopsy was performed, and tissue pathology showed poorly differentiated adenocarcinoma, with signet ring cells. The diagnosis of recurrent colon cancer was therefore made after 19 months after the first diagnosis. WES identified 3 candidate variants from the tissue samples, c.757dupA [p.(Thr253Asnfs*11)] (Tier III) in *TP53*, c.1082G>A [p.(Thr253Asnfs*11)] (Tier III) in *SMAD4*, and c.1082G>A [p.(Thr253Asnfs*11)] (Tier III) in *GNAS*.

Patient 4 was a 6-year-old boy with a recurrent Wilms’ tumor. A palpable abdominal mass was noted at the age of 5 years. A whole abdominal CT scan showed a right renal mass with internal calcification. The patient underwent exploratory laparotomy with tumor removal. Wilms’ tumor stage III was diagnosed, and chemotherapy was given. The tissue pathology revealed a nephroblastoma with favorable histology. After 6 months of follow-up, the patient developed severe abdominal pain, nausea, and vomiting. An abdominal CT scan showed an increase in tumor size with intestinal obstruction. WES of the tissue samples revealed a heterozygous variant, c.96+1G>C in *TP53* (Tier III).

## Discussion

We studied 4 pediatric patients with relapsed or refractory cancer. With WES in tumor–normal pairs of patients’ tissues, we successfully identified significant somatic variants in all cases. Genetic alterations have been demonstrated to be associated with disease progression and prognosis, which could explain the clinical courses of our patients. The findings could be useful for designing experiments to develop novel therapeutic targets.

Patient 1 had relapsed ALL. WES performed in the bone marrow samples identified the variants, c.5890_5906del [p.(Ser1964Leufs*57)] in the *NCOR2* gene. The NM_006312.6 (*NCOR2*): c.5890_5906del [p.(Ser1964Leufs*57)] variant resulted in a frameshift, predicted to cause loss of function (LoF) through protein truncation. The p.S1964Lfs*57 variant is located 1,474 base pairs upstream from the penultimate exon junction of *NCOR2*. The nuclear receptor corepressor 2 (*NCOR2*) encodes proteins that function in B cell development pathways, which include *STAT5*, *FOFOX1*, and *p53*. Lee et al. [7] have reported on the role of NCOR in genomic integrity and transformation. *NCOR2* defects increase the expression of *FOFOX1* and *RAG*, which leads to genomic instability in B-cell ALL. This finding could explain patient phenotypes. Further experiments are needed to verify the effect of the *NCOR2* variant on tumor progression.

Patient 2 was affected with recurrent osteosarcoma. WES of the tissue samples identified the variant, c.7475C>T [p.(Ser2492Leu)] in *COL6A3*. This variant is a missense located in exon 36 of *COL6A3*. There is a large physicochemical difference between serine and leucine, which is likely to impact the secondary protein structure as these residues differ in polarity, charge, and size. The p.Ser2492Leu variant occurs in 4 samples in COSMIC. This missense variant is predicted to be damaging by both SIFT and PolyPhen2. The nucleotide c.7475 in *COL6A3* is predicted to be conserved by GERP++ and PhyloP across 100 vertebrates. The p.Ser2492Leu variant also occurred in an active binding site. This variant is therefore classified as likely oncogenic.

Patient 3 was diagnosed with recurrent colon cancer. WES performed in the tissue samples identified 3 variants, c.757dupA [p.(Thr253Asnfs*11)] (Tier III) in *TP53*, c.1082G>A [p.(Arg361His)] (Tier III) in *SMAD4*, and c.1082G>A [p.(Arg361His)] (Tier III) in *GNAS*. The NM_000546.6 (TP53):c.757dupA [p.(Thr253Asnfs*11)] variant is a frameshift, which is predicted to cause loss of protein function through protein truncation. The p.(Thr253Asnfs*11) variant occurred in 4 samples in COSMIC. The p.(Thr253Asnfs*11) variant has been previously classified as pathogenic in ClinVar. The p.(Thr253Asnfs*11) variant is 343 base pairs upstream from the penultimate exon junction of *TP53*. There are 113 downstream pathogenic LoF variants, with the furthest variant being 142 residues downstream of the variant, p.(Thr253Asnfs*11). The p.(Thr253Asnfs*11) variant results in a LoF of the *TP53* gene, as indicated by the following supporting evidence: the presence of 9 overlapping LoF regions in CiVIC, the presence of existing pathogenic LoF variant NM_000546.6:c.−29+1G>C and 415 others, and 18.76% of variants in *TP53* are LoF in COSMIC. It is therefore classified as oncogenic. *TP53* mutations occur in approximately 40%–50% of sporadic CRC [8]. The development of CRC involves the activation of oncogenes and the inactivation of tumor suppressor genes. *TP53* is a key tumor suppressor gene [9]. *TP53* mutations in CRC occur in 34% of proximal colon tumors and 45% of distal colorectal tumors [10, 11] and are closely related to the progression and outcome of sporadic CRC. Common mutations occur in the DNA binding domain at the hotspot codons, which include 175, 245, 248, 273, and 282 causing the disruption of specific DNA binding and sequential transactivation [12, 13]. The most common *TP53* mutations are at codon p.Arg248 (8%) and at Arg273 (6%) [14]. The NM_005359.6 (SMAD4):c.1082G>A [p.(Arg361His)] variant is a missense in exon 9 of *SMAD4*. There is a small physicochemical difference between arginine and histidine, which is not likely to impact the secondary protein structure as these residues share similar properties. The p.(Arg361His) variant occurs in 183 samples in COSMIC. The p.(Arg361His) variant has been previously classified as pathogenic in ClinVar.

Somatic activating mutations in *GNAS* have rarely been identified in the setting of colorectal adenocarcinoma. A higher proportion of *GNAS* mutations have been reported in tubulovillous and villous adenomas, but no *GNAS* mutations have been identified in tubular adenomas. These data suggest that *GNAS* may function as an important driver mutation during a certain phase of colorectal carcinogenesis, but then could be lost once the biological advantage provided by the mutated gene is no longer needed [15,16,17]. *GNAS* mutations are frequently synchronous with activating mutations in either the *KRAS* or *BRAF* oncogenes [18]. The prognostic and therapeutic significance of *GNAS* mutations in the setting of colorectal adenocarcinoma is currently unknown. *GNAS* mutations have been reported in up to 3% of colorectal carcinomas, most exhibiting a villous morphology [15, 16].

WES of the tissue samples obtained from patient 4 revealed a heterozygous variant, c.96+1G>C in *TP53* (Tier III). The NM_000546.6 (TP53):c.96+1G>C variant affects a splice–donor sequence, potentially resulting in the retention of large segments of intronic DNA by the mRNA and non-functional proteins. The c.96+1G>C variant occurs in 2 samples in COSMIC. The c.96+1G>C variant has been previously classified as pathogenic in ClinVar. The c.96+1G>C variant is predicted to disrupt the existing donor splice site at this position by 3 of 4 splice site algorithms. There are 347 downstream pathogenic LoF variants, with the furthest variant being 395 residues downstream of the variant c.96+1G>C. The c.96+1G>C variant is a LoF variant in the gene *TP53*, which is intolerant of LoF variants, as indicated by the following supporting evidence: presence of 9 overlapping LoF regions in CiVIC, presence of the existing pathogenic LoF variant NM_000546.6:c.−29+1G>C and 415 others, and 18.76% of variants in *TP53* are LoF in COSMIC. For these reasons, this variant has been classified as oncogenic. *TP53* deletions occur in approximately <1% of cancers. Inactivation of *TP53* plays an important role in neoplastic transformation in solid tumors and it has also been reported in hematological malignancies in association with the progression of disease [19,20,21]. Deletion of the *TP53* tumor suppressor gene occurs in approximately 50% of all human cancers [22].

WES has limitations in detecting copy number variants, large gene deletions, large structural variants, and variants located deep within introns. These genetic alterations also play an important role in cancer progression and aggressiveness. Larger studies are required to assess the molecular signature of relapsed or refractory tumors.

This is the first study of WES in tumor–normal pair samples in Thailand. There have been studies of WES in newly diagnosed tumors compared to relapsed/recurrent tumors, both sharing similar mechanisms and variants. In a recent extensive cohort, *TP53* and *PIK3CA* were identified as the most prevalent small gene alterations in somatic tissue. Additionally, copy number variations (CNVs) and fusion genes, such as the EWSR1/FLI1 fusion gene in Ewing sarcoma, play a crucial role in tumorigenesis. Larger studies are imperative to unveil molecular signatures and tumor-specific variants across various types of tumors in the Thai pediatric population.

In conclusion, using WES in tumor–normal pairs of patients’ samples, this study successfully identified somatic variants in all Thai pediatric cases with relapsed or refractory cancers. These findings could provide further insight into the mechanism underlying hard-to-treat cancers.
